# EEG-fNIRS-based hybrid image construction and classification using CNN-LSTM

**DOI:** 10.3389/fnbot.2022.873239

**Published:** 2022-08-31

**Authors:** Nabeeha Ehsan Mughal, Muhammad Jawad Khan, Khurram Khalil, Kashif Javed, Hasan Sajid, Noman Naseer, Usman Ghafoor, Keum-Shik Hong

**Affiliations:** ^1^School of Mechanical and Manufacturing Engineering, National University of Sciences and Technology (NUST), Islamabad, Pakistan; ^2^National Center of Artificial Intelligence (NCAI) – NUST, Islamabad, Pakistan; ^3^Department of Mechatronics and Biomedical Engineering, Air University, Islamabad, Pakistan; ^4^School of Mechanical Engineering, Pusan National University, Busan, South Korea

**Keywords:** recurrence plots (RP), convolutional neural networks (CNN), time distributional layers, long-short term memory (LSTM), brain computer interface (BCI)

## Abstract

The constantly evolving human–machine interaction and advancement in sociotechnical systems have made it essential to analyze vital human factors such as mental workload, vigilance, fatigue, and stress by monitoring brain states for optimum performance and human safety. Similarly, brain signals have become paramount for rehabilitation and assistive purposes in fields such as brain–computer interface (BCI) and closed-loop neuromodulation for neurological disorders and motor disabilities. The complexity, non-stationary nature, and low signal-to-noise ratio of brain signals pose significant challenges for researchers to design robust and reliable BCI systems to accurately detect meaningful changes in brain states outside the laboratory environment. Different neuroimaging modalities are used in hybrid settings to enhance accuracy, increase control commands, and decrease the time required for brain activity detection. Functional near-infrared spectroscopy (fNIRS) and electroencephalography (EEG) measure the hemodynamic and electrical activity of the brain with a good spatial and temporal resolution, respectively. However, in hybrid settings, where both modalities enhance the output performance of BCI, their data compatibility due to the huge discrepancy between their sampling rate and the number of channels remains a challenge for real-time BCI applications. Traditional methods, such as downsampling and channel selection, result in important information loss while making both modalities compatible. In this study, we present a novel recurrence plot (RP)-based time-distributed convolutional neural network and long short-term memory (CNN-LSTM) algorithm for the integrated classification of fNIRS EEG for hybrid BCI applications. The acquired brain signals are first projected into a non-linear dimension with RPs and fed into the CNN to extract essential features without performing any downsampling. Then, LSTM is used to learn the chronological features and time-dependence relation to detect brain activity. The average accuracies achieved with the proposed model were 78.44% for fNIRS, 86.24% for EEG, and 88.41% for hybrid EEG-fNIRS BCI. Moreover, the maximum accuracies achieved were 85.9, 88.1, and 92.4%, respectively. The results confirm the viability of the RP-based deep-learning algorithm for successful BCI systems.

## Introduction

Brain–computer interfaces (BCIs) have become an indispensable element for individuals with disabilities. They have become integral components of new medical applications and have been increasingly applied in communication systems, human–machine interfaces (Bai et al., 2020), and neurofeedback applications (Mercado et al., 2021). BCI enables communication between the human brain and the external computer/device through generated brain commands, thereby avoiding the peripheral nervous system (Antonietti et al., 2021). Moreover, BCI is a neurofeedback method that can enhance the quality of life of patients suffering from serious motor debilities due to tetraplegia (Benaroch et al., 2021), stroke (Mane et al., 2020), and other spinal cord injuries (Al-Taleb et al., 2019). BCI also has applications in neurorehabilitation, communication and control, motor therapy and recovery, brain monitoring, and neuro-ergonomics (Asgher et al., 2020a,b; Mughal et al., 2021). The BCI analyzes a biosignal measured from a healthy subject to predict some intangible aspects of their cognitive state. This process usually consists of three main steps: data acquisition from the brain depending on the application and modality chosen, interpretation or pre-processing data into commands, and output to the computer to generate a command. Among the three types of BCI, namely, reactive, active, and passive BCI (pBCI), pBCI is an important research area that estimates human emotions, cognition, intentions, and behavior based on generated brain responses to different situations.

The demand for improved traditional BCI practices has increased with advances in neuroimaging modalities. Primary non-invasive neuroimaging modalities for BCI include functional magnetic resonance imaging (fMRI), electroencephalography (EEG), magnetoencephalography, and functional near-infrared spectroscopy (fNIRS). Among them, EEG and fNIRS are the foremost modalities in terms of cost and manageability (Rahman et al., 2020; Rashid et al., 2020). EEG measures brain activity by calculating the voltage fluctuations from the action potentials of neurons, whereas fNIRS detects brain activity related to hemodynamic response changes (Hong and Zafar, 2018; Liu et al., 2021). Although invasive techniques provide more accurate data than non-invasive techniques, non-invasive modalities are more frequent and appreciated in the research domain. Non-invasive recording techniques for brain activity improve safety and reduce ethical concerns (Burwell et al., 2017; Pham et al., 2018). Over time, various non-invasive techniques have been used in studies. The most commonly used are EEG, fNIRS, electrooculography, and fMRI (Choi et al., 2017). The selection of a non-invasive modality depends on many factors. Usually, the following parameters are considered: cost, ease of use, and temporal and spatial resolution, as needed by the application. Each modality offers some advantages over the others, and there are always some associated trade-offs; the pros of one modality compensate for the cons of the other modality. Thus, hybrid approaches have proven to be more efficient. Hybrid neuroimaging modalities increase accuracy and offer a greater degree of reasonable control (Hong and Khan, 2017; Khan and Hong, 2017; Hong et al., 2018).

Researchers appreciate the use of low-cost neuroimaging modalities (Hong et al., 2020). Modalities that offer convenience for non-laboratory setups are also choices of interest. In this regard, EEG and fNIRS are the most commonly used. Both are portable and inexpensive compared to the alternatives. Electrodes capture EEG signals due to variations in the current generated by neurons due to postsynaptic activities (Sazgar and Young, 2019). Several electrodes are placed on the subject's scalp for EEG data acquisition. Although EEG provides better temporal resolution ranging up to ~0.05 s, it provides a spatial resolution of only ~10 mm (Puce and Hämäläinen, 2017; Fu et al., 2020). The contrasting comparison of the temporal and spatial resolutions manifests trade-offs when using the EEG modality. In contrast to EEG, fNIRS is an optical imaging technique that measures light absorbance to calculate concentration changes in oxy-hemoglobin and deoxy-hemoglobin within the brain. Similar to EEG, fNIRS is cost-effective and portable. However, unlike EEG, fNIRS provides better spatial resolution. Moreover, fNIRS is less influenced by electrical noise (Hasan et al., 2020; Ghafoor et al., 2022). As evidenced by the comparison, fNIRS can compensate for the trade-offs of EEG. Thus, the EEG and fNIRS hybrid method serve as a breakthrough in neuroimaging (Ahn and Jun, 2017) on theoretical grounds.

As fNIRS measures hemodynamic responses, there is an innate delay in the measurement (Saeed et al., 2020). Various methods have been proposed to compensate for this slow command generation. In this regard, a hybrid method comprising EEG and fNIRS techniques can be used, which proceeds by measuring the initial dip [i.e., at the onset of neural firing, the oxygenated hemoglobin (HBO) level first decreases] instead of the actual hemodynamic response (Hong and Khan, 2017; Kamran et al., 2018). The other contrasting difference between the two modalities is the rate at which data are sampled. The EEG data acquisition rate is ~10–100 times faster than that of fNIRS. When the EEG and fNIRS hybrid is used, it is common to downsample the EEG data to make its processing compatible with that of the fNIRS data (Khan and Hasan, 2020; Ortega et al., 2020). Downsampling might discard some segments of valuable data. As EEG signals are prone to electrical noise, fNIRS suffer from physiological noise, instrumentation, and experimental errors. The experimental errors may be spontaneous, unintentional diversions from the intended protocol, such as motion artifacts or changes in the light intensity in the ambiance. The motion artifacts present in the data can be significantly reduced via Wiener filtering-based methods (Jiang et al., 2019) or wavelet analysis-based methods (Islam et al., 2021). Instrumentation can also induce noise in the data, such as noise from the hardware. However, these noise signals are high-frequency components; thus, they can be eliminated using a low-pass filter. Physiological noises can arise due to breathing activity or heartbeats. Although these noises are unavoidable, many methods have been reported to counter these noises; commonly used techniques apply bandpass filters, parameter mapping, and independent component analysis (Rejer and Cieszyński, 2019; Vourvopoulos et al., 2019; Wankhade and Chorage, 2021). Denoising the data further removes data regions; thus, the processed data are even smaller in magnitude than the raw data. Therefore, the downsampling of the EEG data after pre-processing to match the fNIRS data removes a considerable amount of valuable information regarding brain activity.

Recurrence quantification analysis (RQA) of RP has become popular in recent years for analyzing brain activity because brain signals are both recurrent and dynamic. RP, in general terms, is a non-linear evaluation method for recurrent and dynamic signals. It is a visualization displaying the recurrent occurrences of states *x*(*n*) of a time signal in phase space. RQA is an analysis technique used to quantify the features of the constructed RP. In the literature, RQA feature analysis has been used in EEG signal detection of epilepsy and Alzheimer's disease, coupling and synchronization in EEG of epileptic discharge, and so on. Cortical function during different sleep stages was also analyzed using RP features. The RQA analysis showed that unique RPs were extracted for different sleep stages (Parro and Valdo, 2018). Several studies have also used artificial neural networks (ANNs) (Torse et al., 2019) and support vector machines (SVMs) (Houshyarifar and Amirani, 2017; Zhao et al., 2021) to classify extracted RQA features. One study used a four-layer ANN for different EEG channels to predict the onset of seizures using RQA measures (Torse et al., 2019).

As machine learning (ML) has rapidly become a state-of-the-art analysis tool, researchers have considered searching for classification features (Park and Jung, 2021). The qualitative aspects of these RPs can be used for classification. Moreover, DNNs are highly efficient training classifiers, resulting in better classification accuracy than ML classifiers (Sattar et al., 2021). However, only a few studies that applied these algorithms in BCI are available (Dehghani et al., 2021; Singh et al., 2021). Only one study used a CNN for the binary classification of epileptic seizures from EEG using RP as images (Gao et al., 2020). The practical application of biological feedback in BCI requires efficient and precise motor activity detection and classification methods. These conventional quantification and feature selection methods and simple ML classifiers face several challenges when implementing real-time BCI. Traditional feature engineering methods involve multiple steps, such as feature extraction, feature selection, finding suitable combinations for various features, and sometimes dimensionality reduction from a comparatively small quantity of data, thus leading to other problems such as overfitting and bias (Asgher et al., 2020b). These inherent constraints hinder adjustments by researchers. Therefore, the initial analysis steps, namely, data mining and pre-processing, are time-consuming.

On the other hand, deep learning (DL) algorithms such as CNNs can be employed in two ways for BCI applications: altering or modifying the CNN algorithm architecture to accommodate the one-dimensional time-series data obtained by the modalities or transforming one-dimensional data into two-dimensional (2D) data to be conveniently input to the CNN. Deep neural networks (DNNs) and other traditional classifiers have also been employed based on fNIRS and EEG signals to recognize three different cognitive states (Huve et al., 2019; Takahashi et al., 2021), electromyography signals classification (Oh and Jo, 2021), control of wearable exoskeleton (Sun et al., 2021), and other control applications (Kim et al., 2021; Li et al., 2021; Yaqub et al., 2021). A similar approach has been used for various other applications, such as controlling robots (Huve et al., 2018), differentiating workloads by analyzing the fNIRS signals, and using deep learning techniques. Shoeibi et al. (2022) used an adaptive neuro-fuzzy interface to detect epileptic seizures from EEG signals. The literature also demonstrates the time-delay neural network for classification purposes. Thyagachandran et al. (2020) used this approach to classify EEG signals; however, the presented model was not sufficiently deep to learn the hierarchical features of the EEG signal. The research that resonated most with our present study is that of Tanveer et al. (2019). The authors investigated deep learning-based BCI to detect driver drowsiness. The output strength of the selected channels was translated into color maps and fed into the CNN classifier as an input. The output color maps were obtained by linear mapping the values from the channel to the color intensity. Recent biosignal analysis techniques have a higher inclination toward non-linear dynamics. One of the most widely used methods is the recurrence plot (RP). The analysis focuses on the repeatability of the time-series states and presents the output in geometric structures, whose topology is analyzed to estimate the characteristics of the dynamics (Nayak et al., 2018; Acharya et al., 2019). In the literature, researchers have also experimented with hybrid CNN-LSTM models for time-series biological signal analysis to detect mental disorders. A study to detect schizophrenia via EEG was carried out by Shoeibi et al. (2021) using different ML and DL models, and a comparison was made between applied algorithms based on their accuracy percentage. Among all ML and DL algorithms, CNN-LSTM proved the best architecture for diagnosing schizophrenia.

This study investigated the RP performance for EEG, fNIRS, and hybrid EEG-fNIRS within a deep convolutional neural network and a long short-term memory (CNN-LSTM) model for neuroimaging brain data for BCI. The obtained RPs of EEG and fNIRS were fed as images into the hybrid CNN-LSTM network for classification. The initial hypothesis of this study was that “The classification accuracy of Hybrid EEG-fNIRS BCI will improve by incorporating all signal information from both modalities using recurrence plots instead of using traditional methods of downsampling EEG signals to make them compatible with fNIRS for hybrid BCI.” The main contributions and novelty of our work are as follows:

(i) Implementing whole EEG and fNIRS signals, without any information loss or downsampling, for Hybrid EEG-fNIRS BCI using recurrence plots.
(ii) Implement the time-distributed CNN-LSTM model for activity detection using EEG and fNIRS recurrence plots for hybrid BCI.

Furthermore, to the best of the authors' knowledge, time-distributional (TD) layers were implemented in a network that was not previously used in the BCI field.

The detailed methodology of this research, the dataset used, RP formation from EEG and fNIRS datasets, and the classification approach used for the four-class classification of constructed RP are detailed in the following sections. The related performance of RP in EEG-BCI, fNIRS-BCI, and hybrid EEG-fNIRS-BCI is discussed, and the study's conclusions are provided.

## Methodology

**Figure 1:**
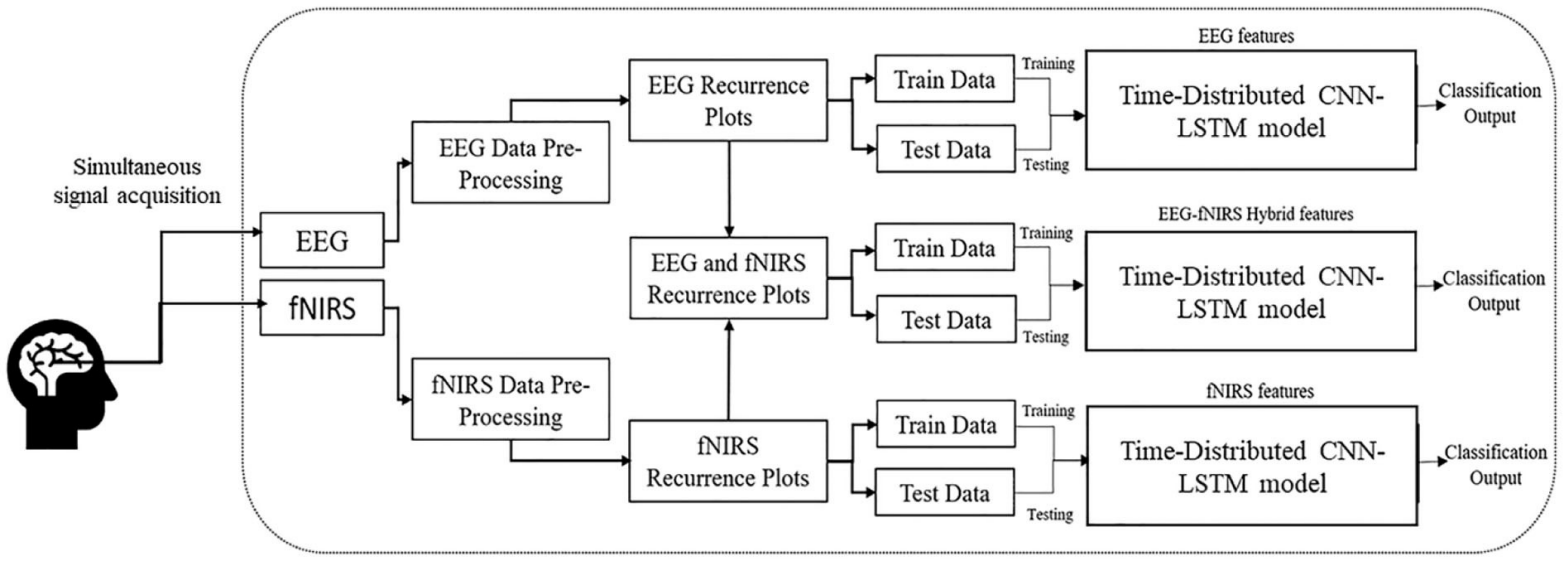
Methodology of the study shows the construction of the hybrid EEG-fNIRS dataset using RPs and classification using time-distributed CNN-LSTM.

In this study, RP performance for EEG, fNIRS, and hybrid EEG-fNIRS with the deep CNN-LSTM model was investigated for neuroimaging brain data for BCI. RP transformed the time series data into the phase space and provided an alternate method to envisage the periodic nature of a time series trajectory, that is, brain signal data in phase space. RPs of EEG and fNIRS were constructed and used as images to feed the hybrid time-distributed CNN-LSTM network for classification. The detailed methodology is described in this section and illustrated in Figure 1.

### Dataset and experimental protocol

**Figure 2:**
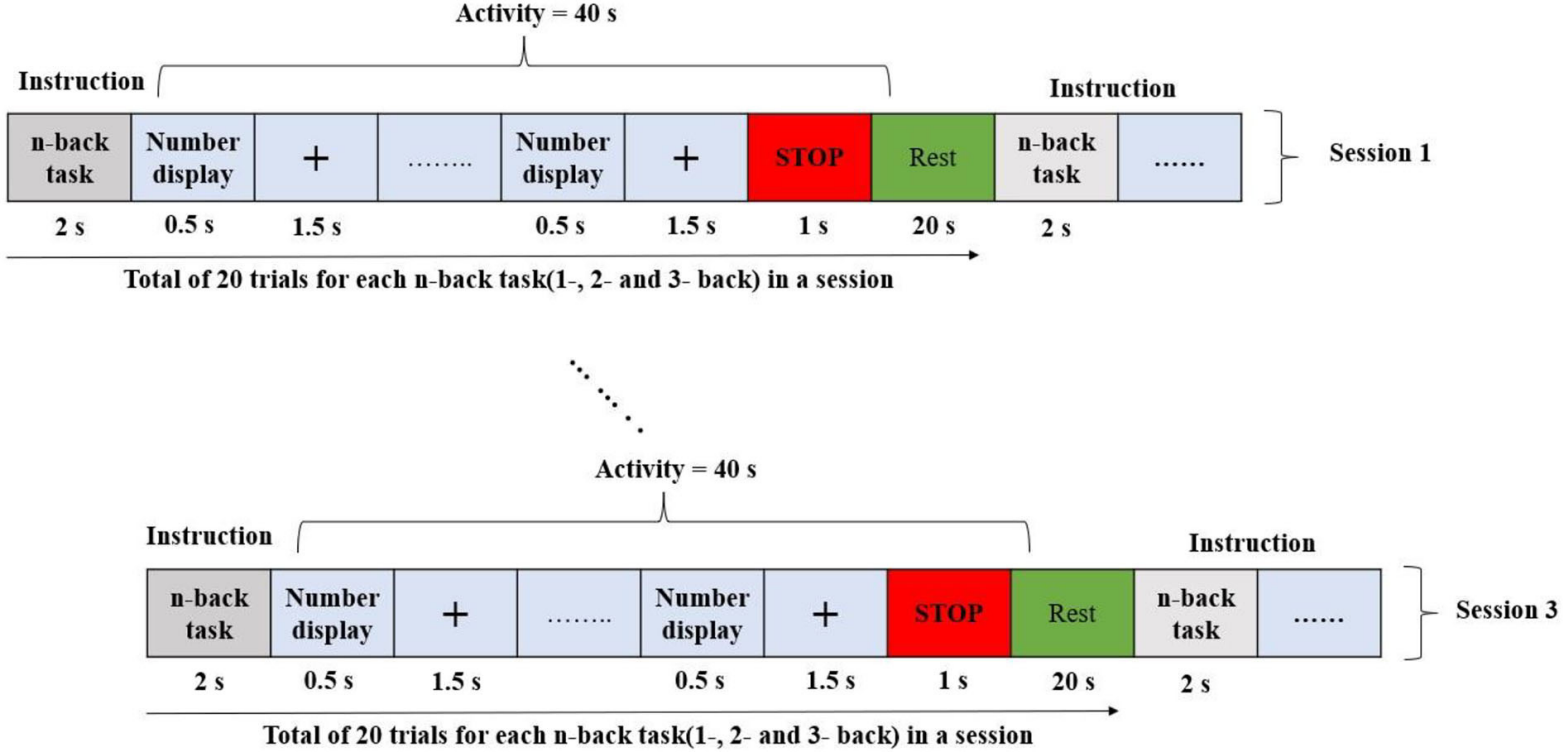
Experiment paradigm of n-back tasks.

The research used an open-source meta-dataset. The data were recorded at the Technische Universität Berlin (Shin et al., 2018) and collected through three different paradigms from 26 healthy participants while focusing on cognitive tasks. Datasets A, B, and C were chosen for the three different cognitive tasks: *n*-back, discrimination response, and word generation, respectively. On these grounds, the selected dataset was an appropriate choice for research in the domain of hybrid BCI. First, task A was performed, followed by tasks C and B. In this study, only dataset A (*n*-back) was used. The entire n-back dataset consisted of three sessions, and each session consisted of three series: 0-back, 2-back, and 3-back tasks. The total recording time for each series was 62 s. The initial 2 s were dedicated to task illustration. The following 40 s were reserved for the task performance (20 numbers displayed for 2 s each on the screen), and the last 20 s were reserved for the rest period. Thus, for every n-task, there were 180 trials. The experimental protocol for the n-back dataset is shown in Figure 2.

### Data acquisition

The EEG and fNIRS data were recorded simultaneously to ensure that the data were synchronized, and a parallel port was used to send the triggers. A BrainAmp EEG amplifier was used to record the EEG data, and the sampling frequency was 1,000 Hz. A stretchable fabric cap was used to place the 30 active electrodes to acquire data in frontal, motor cortex, parietal, and occipital regions according to the internationally recognized 10–5 system (Shin et al., 2018).

**Figure 3:**
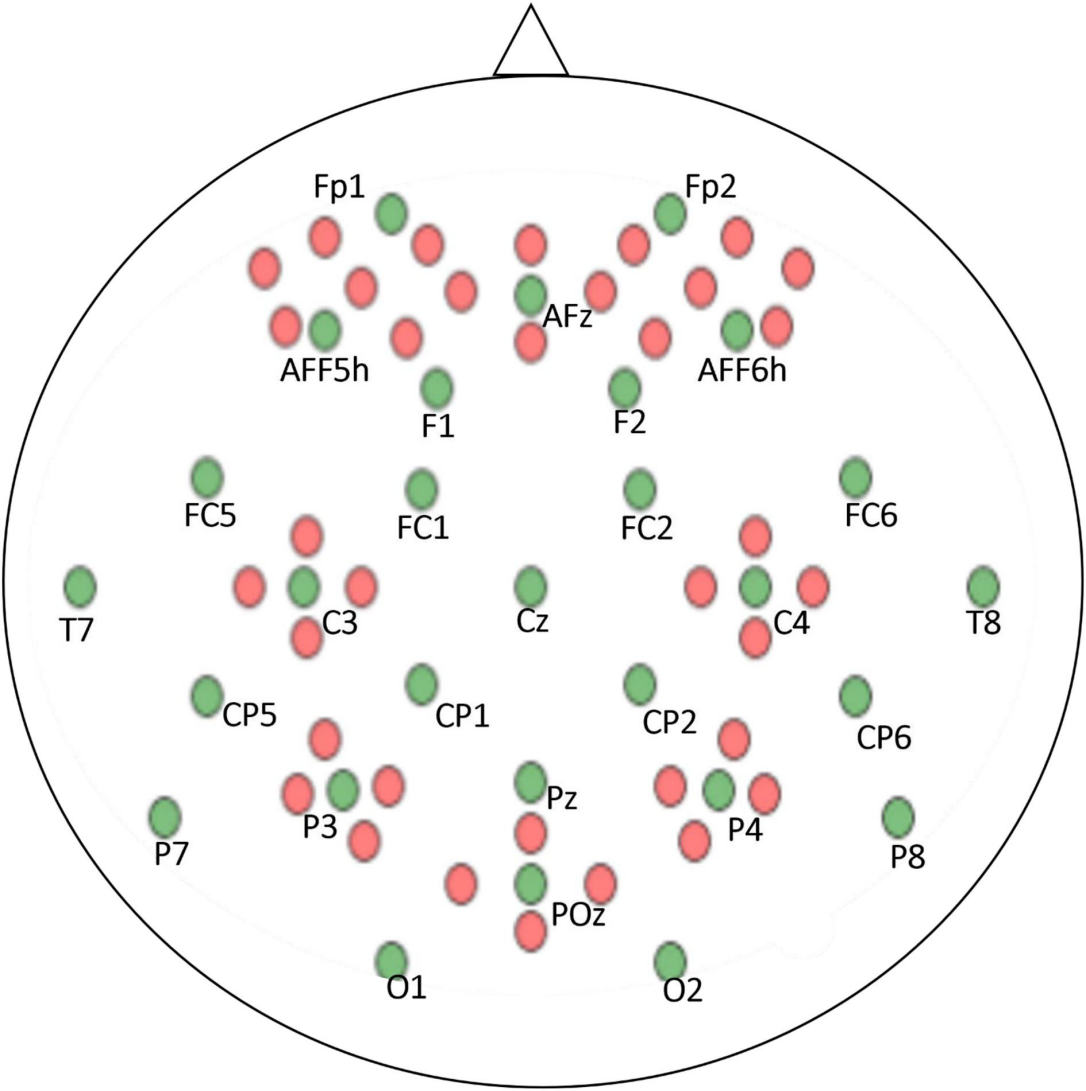
EEG and NIRS electrode positions according to the 10–5 system. Green dots represent EEG electrodes and red dots denote NIRS Optodes.

The fNIRS data were recorded at a sampling frequency of 10.4 Hz via NIRScout (NIRx Medizintechnik GmbH, Berlin, Germany). Sixteen electrodes, representing a combination of sources with detectors, were positioned at the frontal lobe, motor cortex, parietal lobe, and occipital lobe. The optodes of the NIRS were fixed with EEG electrodes on the same cap. The positioning of the electrodes and optodes is illustrated in Figure 3 green circles: EEG electrodes, red circles: NIRS optodes.

### Data pre-processing and labeling

The EEG data were downsampled to 200 Hz. A 6th-order Butterworth bandpass filter with a passband frequency range of 1–40 Hz was used for filtering purposes. The acquired data were first translated into oxy- and deoxy-hemoglobin intensity variations to pre-process the fNIRS data. The conversions were conducted using the modified Beer–Lambert law. The fNIRS raw data were downsampled at 10 Hz. As the fundamental frequency of this dataset was very low, the downsampled data were not fed into the Butterworth bandpass filter. Instead, the data were low-pass filtered to avoid losing the fundamental frequency component. The cutoff frequency of the filter was chosen to be 0.2 Hz. The data were acquired using MATLAB R2013b software. Further processing was performed using Python on Spyder in the Anaconda development environment. After filtration, the dataset was labeled using the activity time markers from the acquired continuous EEG and fNIRS signals. Four classes, namely, 0-, 2-, and 3-back classes, and one of the remaining states, were labeled concerning the experimental protocol. After that, the labeled data were used for RP construction.

### Recurrence plots

A recurrence plot (RP) is a contemporary technique for analyzing non-linear data. This technique employs the visualization of a square matrix whose elements link to the dynamic state repetition. The ordered pair of matrices corresponds to the specific timing of the repetition. Recurrence analysis is a graphical technique that aims to identify hidden recurring patterns (Ledesma-Ramirez et al., 2020). To illustrate this idea, our desired information is univariate time series data and that the data under analysis are a subpart of the large n-dimensional dataset. The topological rendering of the original n-dimensional dataset can be obtained using a single observable variable.

Thus, the embedded matrix, namely, *x*^m^, can be constructed as follows:


(1) $$\begin{array}{l} \left[x_{i}^{m}=\left(x_{i},x_{i+d},x_{i+2d},.......,x_{i+\left(m-1\right)d}\right)\right] \end{array}$$

where *x*_*i*_ is a scalar series, the dimension is represented by *m*, and *d* is the delay. In case the condition


(2) $$\begin{array}{l} m\geq 2n+1 \end{array}$$

is satisfied, the single output variable exhibits the potential to recreate the entire system. Recreation heavily depends on the sequence of the embedded matrix. The sequence can be controlled by adequately choosing parameters *m* and *d*. The asymmetric matrix of the Euclidean distances can also be constructed by measuring the distance between pairs of embedded vectors. These distances are translated into an equivalent color, and each distance has a distinctive color. Thus, an RP is a square assortment of pixels whose color depends on the corresponding magnitude of values. The pixel coordinates also carry useful information that is linearly related to the location of the data in the original data matrix.

The use of ε is commonly employed in RPs. This ε is referred to as the critical radius. Each value is compared with the critical radius to check whether the pixel value is ≤ε; then, the pixel is displayed as a darkened pixel. In other words, RP is a visualization of a square recurrence matrix showing all the instances of times at which a state of a non-linear system repeats; the columns and axes of the recurrence matrix correspond to specific time intervals. In technical terms, an RP shows every time of a non-linear time signal from a dynamical system at which its phase space trajectory spans approximately the same area in the phase space. In graphical terms, this is a graph of


(3) $$\begin{array}{l} \vec{x}\left(i\right)\approx \vec{x}\left(j\right) \end{array}$$

where *i* is on the horizontal axis, *j* is on the vertical axis, and $\vec{x}$ is the phase space trajectory of the dynamical system. Thus, a binary recurrence matrix is constructed using a specific time window *w* = 5 s, where any two-time steps are separated by the time interval ε = 0.1 and a step size of 10 in the following manner:


(4) $$R(i,j)=\left\{ \begin{array}{l} 1\;if\;||\vec{x}(i)-\;\vec{x}(j)||\;\leq \;\varepsilon \;\\ 0\;otherwise \end{array}\right.$$

where *i* and *j* are the horizontal and vertical time axes, *i, j* ϵ {*t*_0_, *t*_1_….*t*, …. *t*_T_}. The RP is a visualization of the recurrence matrix with a black square of the lattice at coordinates (*i, j*) if *R*(*i, j*) = 1 and a white square if *R*(*i, j*) = 0.

**Figure 4:**
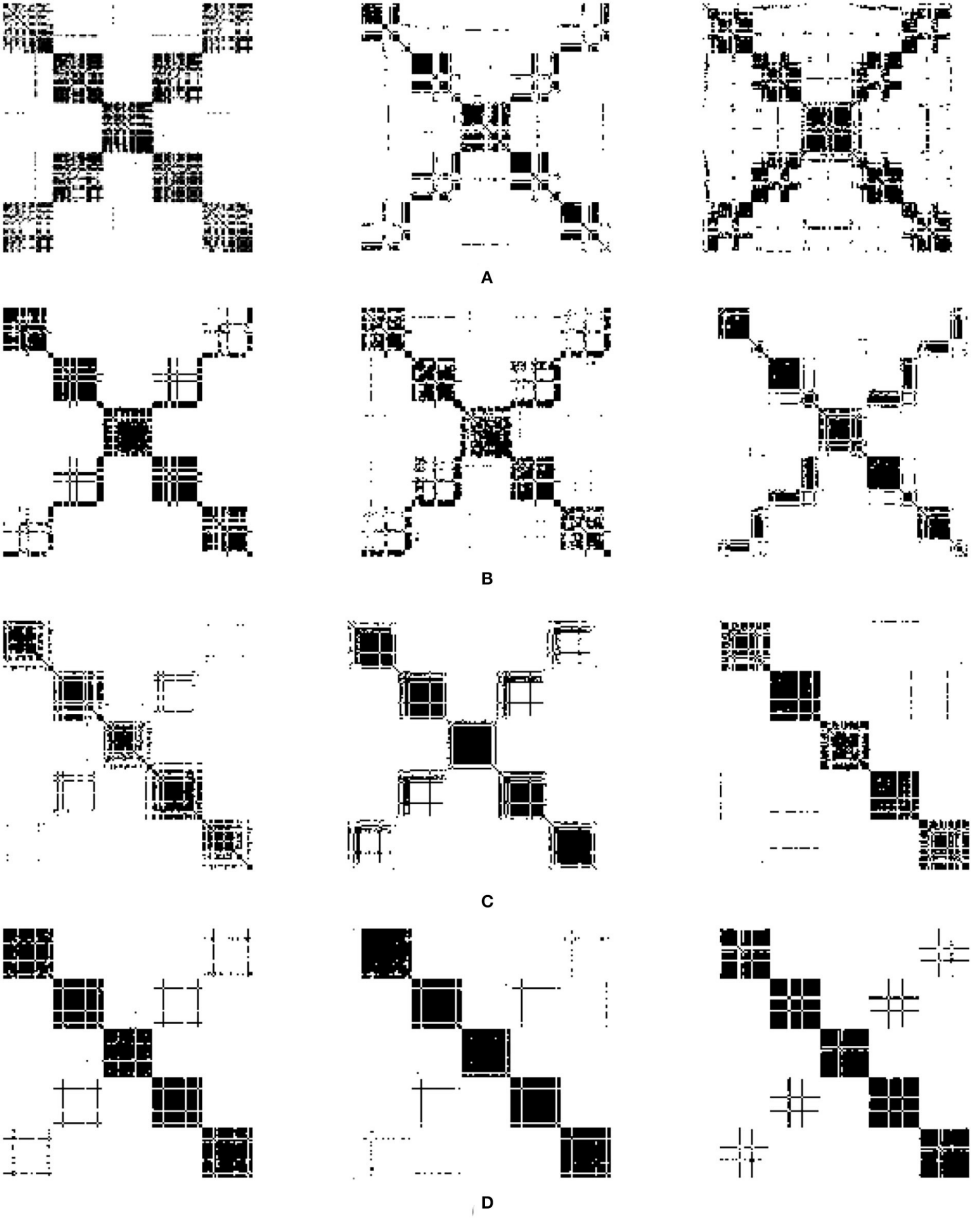
RPs of fNIRS dataset. **(A)** 0-back, **(B)** 2-back, **(C)** 3-back, **(D)** rest.

Figure 4 shows the corresponding RPs constructed using the fNIRS dataset for the 0-, 2-, and 3-back classes, and the rest state for Subject 1.

After experimenting with different values for the parameters of the RPs, ε = 0.1 and step size = 10 were adopted. Figure 4 depicts the non-linear mapping of the acquired brain signals to the new dimension through the RPs with the selected parameters. Each subject's RP data were split into training and test datasets using 10-fold cross-validation before performing classification to avoid overfitting and provide better generalization. Moreover, the model performance was evaluated based on the following performance metrics.

#### Accuracy

The accuracy of a classifier is the proportion of the total number of correct predictions made by the classifier. If the confusion matrix is given, accuracy is defined as:


$$\begin{array}{l} Accuracy\;=\;\frac{True\;Positives\;+\;True\;Negatives}{Total\;Number\;of\;Samples} \end{array}$$

#### Precision

Precision or positive predictive value is the proportion of the correctly predicted positive cases of all cases predicted positively by a classifier. Given a confusion matrix, precision is defined as:


$$\begin{array}{l} Precision\;=\;\frac{True\;Positives}{Total\;Number\;of\;Samples\;Predicted\;as\;Positive} \end{array}$$

#### Recall

Recall or sensitivity is the proportion of all actual positive cases that were correctly predicted to be positive by a classifier. Given a confusion matrix, recall is defined as:


$$\begin{array}{l} Recall\;=\;\frac{True\;Positives}{Total\;Number\;of\;Actual\;Positive\;Samples} \end{array}$$

#### F1 score

The F1 score is the harmonic mean of precision and recall values for a given classification model. Given a confusion matrix, the F1 score is defined as:


$$\begin{array}{l} Precision={\left(\frac{Precision^{-1}+\;Recall^{-1}}{2}\right)}^{-1} \end{array}$$

### Time distributed CNN-LSTM

A CNN is a multilayered neural network with architecture to detect complex features in the data. Unlike traditional multilayer perceptron architectures, CNN uses two operations called “convolution” and “pooling” to reduce the image into its essential features, which are used to understand and classify it. CNNs are composed of basic building blocks, which include the following: a convolutional layer with a filter or kernel passed over an image; an activation layer that usually has an activation function; a rectified linear unit (ReLU) to introduce non-linearity that allows the network to train itself through backpropagation; a pooling layer that downsamples and reduces the size of the matrix and is focused on the most prominent information in each feature of the image; and a fully connected layer that outputs the different probabilities associated with every label attached to the image. The label with the highest probability is the classification decision. CNNs are widely used in agriculture, self-driving vehicles, healthcare, and surveillance. LSTM networks are recurrent neural networks (RNNs) that use special and standard units. The special units include the “memory cell,” which maintains information in its memory for longer. LSTM has feedback connections, unlike standard feed-forward neural networks; it can process entire data sequences, including speech and video. LSTM is widely used in speech recognition, handwriting recognition, handwriting generation, music generation, language translation, image captioning, and anomaly detection in intrusion detection systems. A simple LSTM unit comprises a cell, input, output, and forget gate. The cell remembers the information, whereas the gates regulate the flow of information. LSTM networks are modified forms of RNNs; they remember past data in memory.

Over time, researchers have applied different architectures and types of deep learning networks. Unlike images, text, voice, and other widely used datasets, neuroimaging signals are intrinsically different and have an important chronological order. This chronological order dictates the flow of information necessary to detect activities or actions. Examples of such chronological order are the initial dip at the start of activity in fNIRS signals and positive deflection in event-related potential P300 signals in EEG. A novel CNN-LSTM network was designed for this study. The network consists of one CNN and one LSTM module combined with a dense layer. After pre-processing, the data are fed into the CNN module; this module consists of two convolutional layers, each with 16 filters and ReLU as the activation function, and one max-pooling layer. CNNs are best known for their abilities of feature extraction from 2D and 3D images. Considering the data sequence used in the form of chronologically ordered time windows, the relationship between two windows in a given input should be detected. An LSTM layer enables the network to use its memory and enhance its prediction. The convoluted output from the CNN block is reshaped and flattened before being fed into the LSTM layer. The layers preceding the LSTM layers are wrapped inside a time-distributed layer that allows their application to every temporal slice of the input data. This time-distributed wrapper applies the same instance of convolutional layers to each timestamp, such that the same set of weights is used. After passing through another dense layer, the LSTM layer terminates into the output layer.

**Figure 5:**
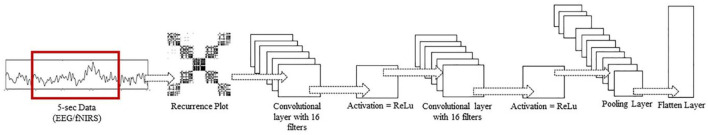
Inside a TD layer. RP input to two Conv2D layers, each with 16 filters, and ReLu as activation function, followed by a max pool and flatten layer.

**Figure 6:**
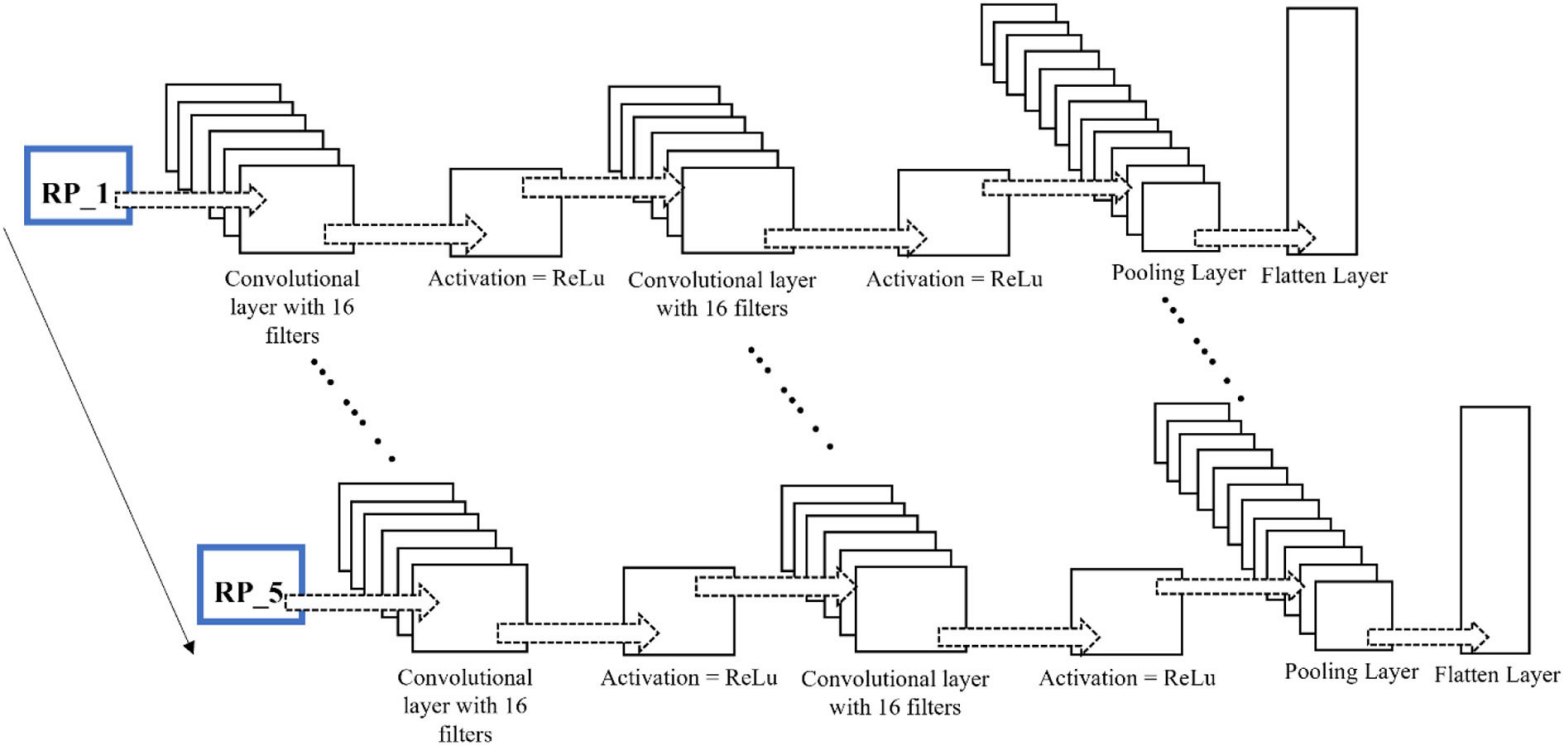
Time-distributed CNN-LSTM network for classification of four-class mental workload using RPs of EEG and fNIRS dataset.

**Figure 7:**
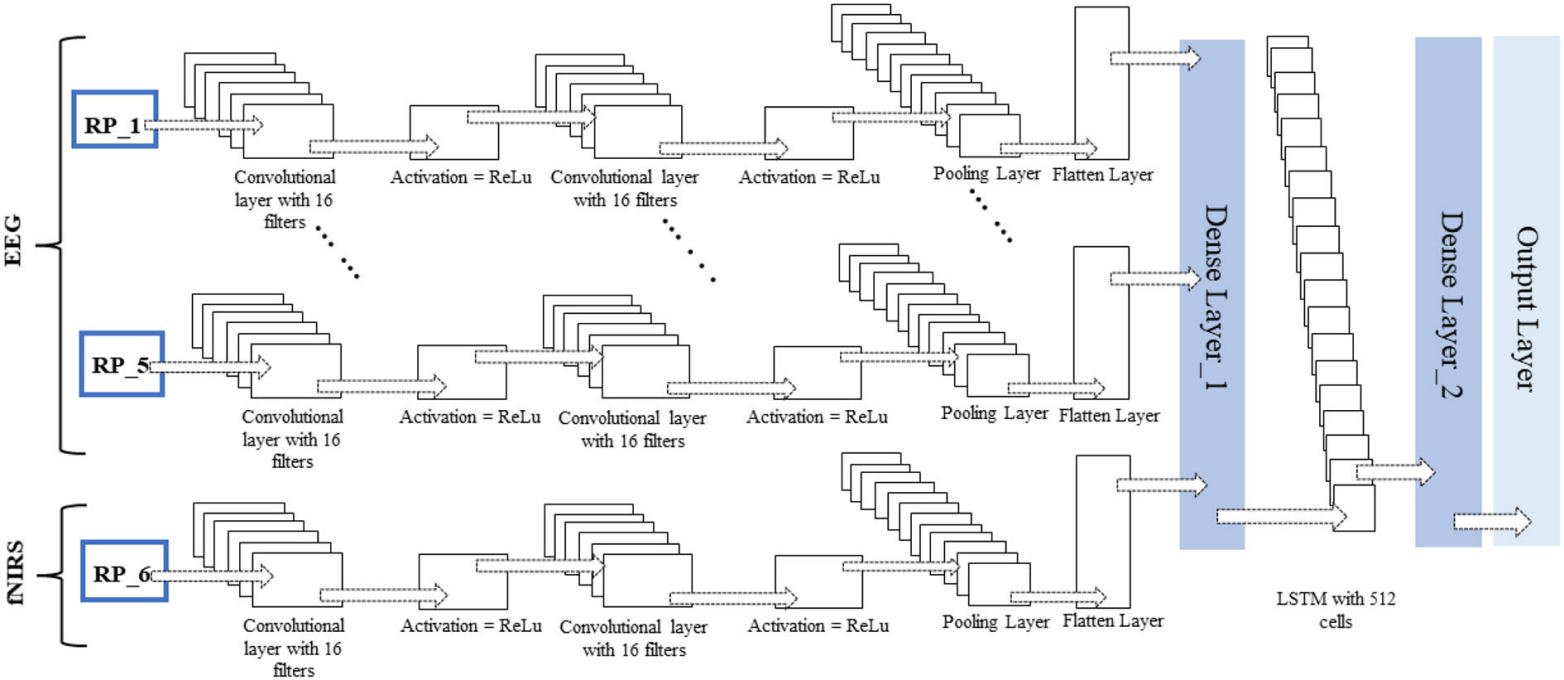
Time-distributed CNN-LSTM network for classification of four-class mental workload using RPs of hybrid EEG-fNIRS dataset.

No researcher has exploited this chronological order using time-distributed layers in deep learning models to the authors' knowledge. The constructed RPs with a fixed window length and an overlapping portion are fed into the network as images. The different configurations of this proposed network for fNIRS, EEG, and hybrid modalities are discussed in detail in the Discussion section. The network architecture for the EEG and fNIRS BCI and the details of the hyperparameters of the DL model used, that is, several layers, dimensions, the number of filters used in each layer, and the number of neurons, among other details, are shown in Figure 7. Researchers have invested tremendous efforts to determine the single best architecture for deep learning neural networks, giving rise to the sub-research field known as neural architecture search (NAS). However, there is no definite answer regarding the optimal neural architecture a priori. The number of neurons, number of filters, number of layers, their combinations, dropout, and max-pooling percentage remain the best hyperparameters. The most viable approach seems to be using intuition and domain knowledge to determine an initial guess for these parameters and then iteratively shortlist them to obtain good values. In this study, the NAS design process was as follows: a network with a minimum number of parameters, a single convolutional layer, a single LSTM layer, and one dense layer was created; other hyperparameters were tuned; more layers were added, and the network hyperparameters were tuned with a grid search using the sklearn wrapper. We performed the above grid search with sample data and chose the best-performing network for EEG, fNIRS, and EEG+fNIRS datasets. However, this approach resulted in input dimension mismatch because of the extra number of features in the hybrid dataset compared to the single modality datasets. We solved this problem by adding another sequence module on top of the EEG network architecture and wrapping it inside the TD layer, similar to the EEG network. The later stages of a network-like dense layer, LSTM layer, and the following layers remained the same; however, this strategy solved the input dimensionality mismatch problem. Figures 5–7 show the network architecture of the study.

## Results

The time-distributed CNN-LSTM was used in this research to classify four classes, namely, the three n-back activities and the rest state from the fNIRS dataset acquired from 26 subjects. The data acquisition and initial pre-processing included filtering EEG data using a zero-phase, low-pass, 6th-order Butterworth filter. In the case of fNIRS, conversion of light densities into changes in the concentrations of HbO and HbR (hemodynamic response) was performed using the modified Beer–Lambert law followed by a zero-phase, low-pass, 6th-order Butterworth filter. After that, the data were labeled along with outlier rejection and data normalization. The window size selection in the hybrid EEG/fNIRS-based BCI system is essential because hemodynamics response has an inherent delay, which requires 0–10 s to complete after the stimulus. In the literature, researchers have experimented with different windows of varying lengths, such as 2–9, 2–7, and 3–7 s (Khan and Hong, 2017; Gaur et al., 2021). Generally, the smaller the window size, the better the BCI performance will be. After an initial investigation, a window size of 5 s with 20% overlap was used for all BCIs. After that, the RPs for segmented signals were constructed into a sequence of 5 s windows.

**Figure 8:**
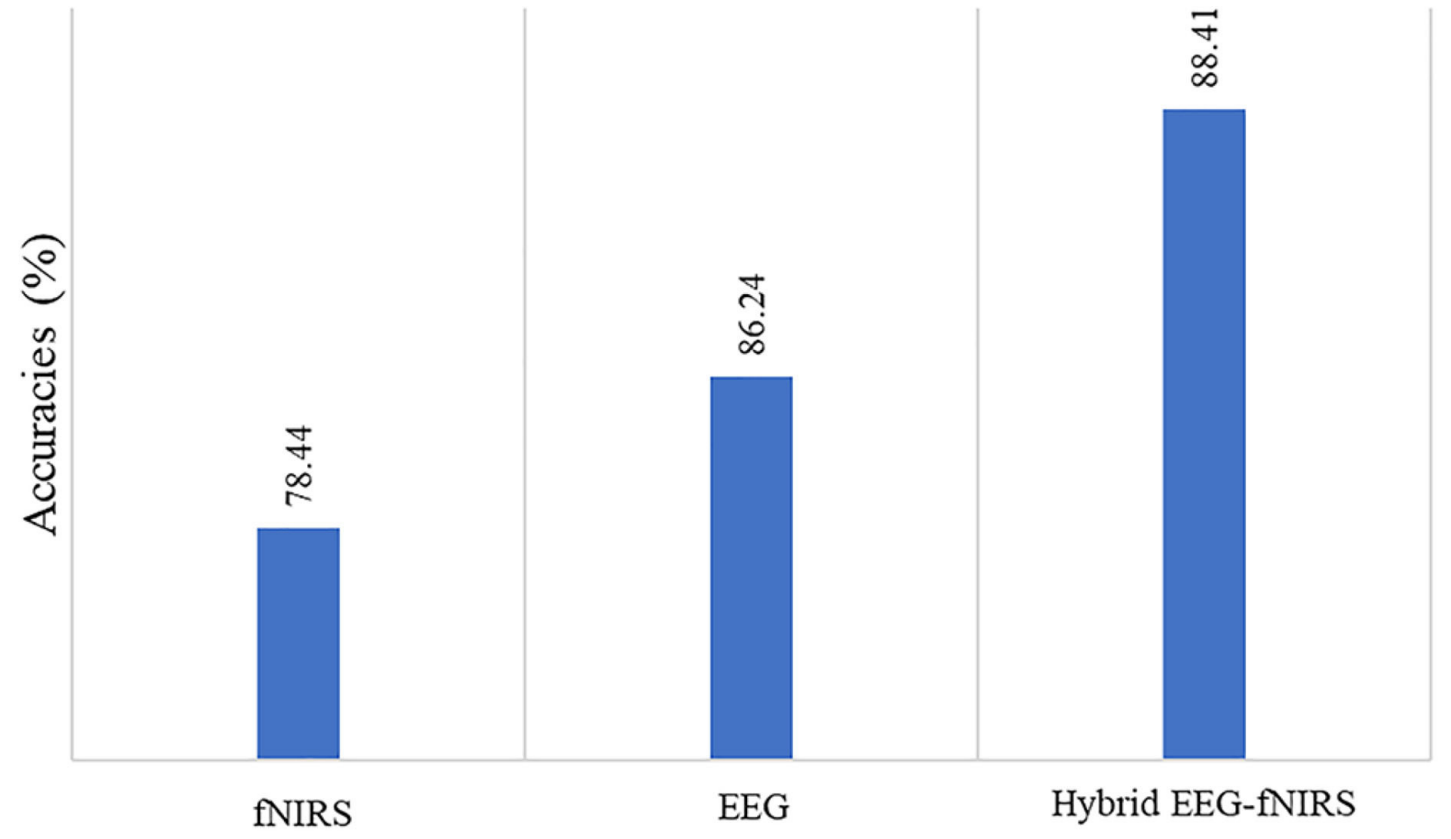
Comparison of accuracies of fNIRS-based BCI, EEG-based BCI, and hybrid EEG-fNIRS-based BCI.

The deep learning algorithms were trained on a GTX 1060 graphic card with 3 GB VRAM and an Intel 6th Gen Core i7-6700HQ processor with a 3.2-GHz frequency. The Keras API was used with the TensorFlow backend on Spyder in the Anaconda integrated development environment. The average accuracy achieved for the four-class classification was 78.4% for fNIRS, 86.44% for EEG, and 88.41% for hybrid EEG-fNIRS BCI. The maximum accuracies achieved were 82.4, 89.75, and 93.58%, respectively. Table 1 summarizes the results of the 26 participants in terms of their classification accuracies, precision, recall, and F1 score for EEG-BCI, fNIRS-BCI, and Hybrid EEG-fNIRS BCI. Figure 8 shows the average accuracies achieved by these approaches.

**Table 1:** Performance evaluation metrices of fNIRS-BCI, EEG-BCI, and hybrid EEG-fNIRS-BCI.

	**S1**				**S2**			
	**Accuracy**	**Precision**	**Recall**	**F-1 score**	**Accuracy**	**Precision**	**Recall**	**F-1 score**
fNIRS-BCI	81.32	81.00	80.66	80.53	81.12	82.20	80.35	80.72
EEG-BCI	85.11	85.41	84.87	84.66	86.34	86.44	86.01	86.00
Hybrid-BCI	89.63	90.09	89.26	89.45	86.78	87.19	86.13	85.90
	**S3**				**S4**			
	**Accuracy**	**Precision**	**Recall**	**F-1 score**	**Accuracy**	**Precision**	**Recall**	**F-1 score**
fNIRS-BCI	77.25	77.80	76.56	76.59	71.74	72.15	70.70	70.52
EEG-BCI	86.53	87.01	86.15	86.11	85.60	85.87	84.76	84.47
Hybrid-BCI	88.33	88.41	88.09	87.91	86.66	86.88	86.61	86.58
	**S5**				**S6**			
	**Accuracy**	**Precision**	**Recall**	**F-1 score**	**Accuracy**	**Precision**	**Recall**	**F-1 score**
fNIRS-BCI	70.57	71.26	69.75	69.82	76.74	78.70	75.79	76.08
EEG-BCI	89.31	89.33	89.05	89.01	85.55	85.22	84.92	84.76
Hybrid-BCI	91.79	92.01	91.36	91.37	89.81	89.57	89.39	89.28
	**S7**				**S8**			
	**Accuracy**	**Precision**	**Recall**	**F-1 Score**	**Accuracy**	**Precision**	**Recall**	**F-1 score**
fNIRS-BCI	77.74	78.53	77.28	76.90	82.44	82.82	82.15	82.10
EEG-BCI	89.00	89.44	88.79	88.84	82.14	82.53	81.89	81.98
Hybrid-BCI	92.28	92.57	92.27	92.28	83.32	83.15	82.76	82.35
	**S9**				**S10**			
	**Accuracy**	**Precision**	**Recall**	**F-1 score**	**Accuracy**	**Precision**	**Recall**	**F-1 score**
fNIRS-BCI	80.40	80.98	79.83	79.84	80.14	80.38	79.52	79.59
EEG-BCI	88.76	89.25	88.61	88.51	84.06	84.70	84.00	84.01
Hybrid-BCI	90.62	90.61	90.28	90.38	87.40	87.90	87.45	87.25
	**S11**				**S12**			
	**Accuracy**	**Precision**	**Recall**	**F-1 Score**	**Accuracy**	**Precision**	**Recall**	**F-1 score**
fNIRS-BCI	79.16	79.67	78.34	78.27	77.18	76.65	76.37	75.85
EEG-BCI	83.93	84.09	83.50	83.53	89.75	89.89	89.56	89.59
Hybrid-BCI	86.71	86.88	86.54	86.51	91.35	91.45	91.18	91.13
	**S13**				**S14**			
	**Accuracy**	**Precision**	**Recall**	**F-1 score**	**Accuracy**	**Precision**	**Recall**	**F-1 score**
fNIRS-BCI	75.15	75.39	74.22	74.16	77.00	79.12	75.66	76.23
EEG-BCI	89.63	89.92	89.10	88.51	87.84	87.57	87.36	87.26
Hybrid-BCI	91.91	92.11	91.73	91.68	89.93	89.90	89.72	89.57
**S15**				**S16**			
	**Accuracy**	**Precision**	**Recall**	**F-1 score**	**Accuracy**	**Precision**	**Recall**	**F-1 score**
fNIRS-BCI	74.64	75.56	73.45	73.47	78.66	79.20	77.80	77.71
EEG-BCI	86.35	87.24	85.97	85.51	82.33	82.61	81.48	81.59
Hybrid-BCI	87.34	87.46	86.76	86.64	83.50	83.59	83.33	82.99
	**S17**				**S18**			
	**Accuracy**	**Precision**	**Recall**	**F-1 score**	**Accuracy**	**Precision**	**Recall**	**F-1 score**
fNIRS-BCI	80.27	80.60	79.60	79.75	81.76	82.07	81.48	81.48
EEG-BCI	87.77	87.88	87.78	87.60	89.68	89.92	89.62	89.49
Hybrid-BCI	92.16	92.83	91.69	91.81	93.58	93.67	93.66	93.56
	**S19**				**S20**			
	**Accuracy**	**Precision**	**Recall**	**F-1 Score**	**Accuracy**	**Precision**	**Recall**	**f-1 Score**
fNIRS-BCI	79.35	80.48	79.55	79.17	80.33	80.86	79.49	79.71
EEG-BCI	86.16	86.08	85.69	85.64	87.52	87.80	86.92	87.01
Hybrid-BCI	87.63	87.84	86.91	87.04	91.36	91.87	91.06	91.11
	**S21**				**S22**			
	**Accuracy**	**Precision**	**Recall**	**F-1 Score**	**Accuracy**	**Precision**	**Recall**	**F-1 Score**
fNIRS-BCI	74.21	75.29	72.88	73.14	82.37	83.42	81.60	81.96
EEG-BCI	87.89	88.04	87.64	87.46	79.91	80.76	78.72	78.81
Hybrid-BCI	89.75	89.95	89.44	89.33	81.65	82.46	81.05	81.11
	**S23**				**S24**			
	**Accuracy**	**Precision**	**Recall**	**f-1 score**	**Accuracy**	**Precision**	**Recall**	**F-1 score**
fNIRS-BCI	79.60	79.75	78.90	78.91	78.61	78.82	78.05	78.12
EEG-BCI	84.37	83.94	83.70	83.62	86.96	87.64	86.52	86.77
Hybrid-BCI	85.36	85.18	84.73	84.46	89.81	90.03	89.70	89.62
	**S25**				**S26**			
	**Accuracy**	**Precision**	**Recall**	**F-1 score**	**Accuracy**	**Precision**	**Recall**	**F-1 score**
fNIRS-BCI	80.27	80.77	79.40	79.47	81.45	81.72	80.56	80.58
EEG-BCI	84.43	85.21	83.81	83.86	85.17	85.15	84.18	84.30
Hybrid-BCI	84.92	85.63	84.57	84.52	85.17	85.62	84.48	84.16

## Discussion

Researchers appreciate the use of low-cost neuroimaging modalities. Modalities that offer convenience to non-laboratory setups are also choices of interest. In this regard, EEG and fNIRS are the most commonly used neuroimaging modalities. Both are portable and inexpensive compared to fMRI. However, EEG offers a spatial resolution of only ~10 mm (Puce and Hämäläinen, 2017; Fu et al., 2020). The contrasting comparison of the temporal and spatial resolutions manifests trade-offs when using the EEG modality.

In contrast to EEG, fNIRS constructs functional neuroimages of the brain by employing NIR light. As fNIRS measures hemodynamic responses, there is an innate delay in the measurement (Saeed et al., 2020). Various methods have been proposed to compensate for this slow command generation. In this regard, hybrid EEG-fNIRS can be an option. However, the sampling frequencies of both modalities are different, thus resulting in information loss. Moreover, the most important objective of all studies conducted on BCI is to enhance real-time classification accuracy and reduce computational costs with multiple commands, thus emphasizing the need to develop appropriate identification and classification methods for real-time BCI (Phanikrishna et al., 2021). Usually, multi-channel brain signal acquisition modalities (i.e., EEG) analyze brain motor activity using different methods, such as time and frequency feature analysis, event-related synchronization-desynchronization analysis, common spatial or temporal patterns, and spatial-spectral decomposition. Most of these methods require high computational costs and are less feasible to use for real-time BCI (Janapati et al., 2020). With advances in brain signal acquisition modalities, the demands for better signal processing and feature extraction have also increased. Traditional methods of extracting useful information from multi-channel brain signal acquisition modalities, such as time and frequency analysis, event-related synchronization and desynchronization analysis, and finding common spatial and temporal patterns are computationally expensive and not very feasible for real-time BCI applications. RQA of RP has become popular in recent years for analyzing brain activity because brain signals are both recurrent and dynamic. RQA is an analysis technique used to quantify features of the constructed RP. In the literature, RQA features have been used in EEG signal detection of epilepsy and Alzheimer's disease, coupling, and synchronization in EEG of epileptic discharge. Cortical function during different sleep stages was analyzed using RP features. The RQA analysis showed that unique RPs were extracted for different sleep stages (Parro and Valdo, 2018). Several studies have also used SVM and ANNs to classify extracted RQA features. One study used a four-layer ANN for different EEG channels to predict the onset of seizures using RQA measures (Torse et al., 2019).

Considering the complexity and computational cost of DNNs, researchers have invested tremendous efforts to determine the best architecture for deep learning neural networks, giving rise to the sub-research field known as NAS. However, there is no definite conclusion regarding the optimal neural architecture a priori. The number of neurons, number of filters, number of layers, their combinations, dropout, and max-pooling percentage remain the best hyperparameters. The most viable approach seems to be using intuition and domain knowledge to determine an initial guess for these parameters and then iteratively shortlist to obtain good values. In this study, the NAS design process was as follows: a network with a minimum number of parameters, a single convolutional layer, a single LSTM layer, and one dense layer was created; other hyperparameters were tuned; more layers were added, and the network hyperparameters were tuned with a grid search using the sklearn wrapper. We performed the above grid search with sample data and chose the best-performing network for the fNIRS dataset. Another advantage of using an RP with a DNN is that it incorporates the entire signal and does not require any extra steps, such as feature extraction and feature selection. Moreover, it also minimizes extra pre-processing steps, such as finding temporal or spatial features. The constructed RPs of EEG and fNIRS were fed to the classification network to detect the class of activity (0, 2-back, 3-back, or rest). The classification network used was the time-distributed CNN-LSTM. CNN is best known for feature extraction from multidimensional images. In contrast, the RNN has an excellent pattern recognition ability for input sequences. However, CNN and RNN have stability issues due to either exploding or vanishing gradients. An RNN variant was used to solve this issue by using memory cells and LSTM. The highest classification accuracy for four-class mental workload data for the BCI was achieved using this network.

The study results indicate that using the hybrid modalities for the classification of BCI results in higher accuracy than that of the single modality, along with an increase in the number of commands and a reduction in detection time. Figure 8 shows the comparison of the average accuracies achieved for all BCIs. The results of our study have proven the initial hypothesis that incorporating the entire EEG signal for hybrid EEG-fNIRS BCI instead of downsampling will significantly increase classification accuracy. The classification accuracies achieved with our proposed methodology were the highest compared to other classification methods for four-class EEG-based BCI and other studies on the same dataset for four-class classification for hybrid EEG-fNIRS. A comparison with relevant studies is presented in Tables 2, 3, respectively. Moreover, implementing the TD layers resulted in faster and easier computation. This may prove to be a state-of-the-art algorithm in the present BCI realm. The results show a promising future for the use of RPs in real-time BCI. The proposed classification method can help improve the accuracy of real-time BCI.

**Table 2:** Comparison with other 4-class classification studies for BCI.

**Authors**	**Brain acquisition modality**	**Classes**	**Subjects**	**Methods**	**Performance %**
Ge et al. (2014)	EEG	4	3	CSP and SVM	72.3, 73.2
Wang et al. (2014)	EEG	4	9	ICA and SVM	71.8
Naeem et al. (2006)	EEG	4	8	ICA and CSP	Between 33 and 84
Our work	fNIRS	4	26	Time distributed CNN-LSTM	78.44
Our work	EEG	4	26	Time distributed CNN-LSTM	86.24
Our work	Hybrid EEG-fNIRS	4	26	Time distributed CNN-LSTM	88.41

**Table 3:** Comparison with other 4-class hybrid classification studies for BCI with same dataset.

**Authors**	**Brain acquisition modality**	**Classes**	**Subjects**	**Methods**	**Performance %**
Saadati et al. (2020)	EEG-fNIRS	4	26	DNN	87
Kwon et al. (2020)	EEG-fNIRS	3	26	CSP	77.6
Our work	Hybrid EEG-fNIRS	4	26	Time distributed CNN-LSTM	88.41

The limitations of our work are as follows: first, the proposed methodology is computationally costly, and substantial computational resources are required to train and test deep learning models with large datasets because the size of RP increases exponentially with the data size fed at a time, as does the model complexity. Second, the proposed algorithm has not yet been implemented for real-time BCI, which leaves room for network improvement and optimization. There are many potential applications for RPs in BCIs other than EEG and fNIRS signals, as all biological signals constitute dynamic time series data, and our study has validated the successful implementation of RP for time-series data analysis. In the future, further work can be conducted to explore and experiment with new deep learning methods in computer vision along with RPs, such as transformers and attention learning for active channel selection for real-time BCI. Working in this direction will help researchers mitigate nuances related to deep learning algorithms in BCI.

## Conclusion

This paper provides an inimitable time-distributed convolutional neural network and long short-term memory method for the integrated categorization of fNIRS-EEG for hybrid BCI applications. The recorded brain signals are first projected onto a non-linear dimension via RPs and supplied into the CNN to extract critical characteristics without downsampling. Then, LSTM is utilized to learn the chronological properties and time-dependence relation to identify brain activity. The average accuracy levels were 78.44% for fNIRS, 86.24% for EEG, and 88.41% for hybrid EEG-fNIRS BCI when using the suggested model. The findings support the RP-based deep-learning algorithm's suitability for effective BCI applications.

## Data availability statement

The original contributions presented in the study are included in the article, further inquiries can be directed to the corresponding author.

## Ethics statement

The original study by Shin et al. (2018) was reviewed and approved. The data set is an open-source meta dataset of Shin et al. (2018). The patients/participants provided their written informed consent to participate in the original study. For the current study, ethical review and approval, and written informed consent, were not required, in accordance with the local legislation and institutional requirements.

## Author contributions

NM wrote the first draft manuscript. MK conceived the idea of the manuscript and supervised the process. KK, KJ, and HS collected and analyzed the methodologies reported in the paper. NN, UG, and K-SH participated in the revision of the manuscript. NM, MK, UG, and K-SH revised and edited the manuscript. All authors contributed to the article and approved the submitted version.

## Funding

This work was supported by the Technology Innovation Program (Grant No. 20016846, Development of fNIRS-based multimonitoring healthcare device for sleep state diagnosis) funded by the Ministry of Trade, Industry, and Energy (MOTIE, Korea).

## Conflict of interest

The authors declare that the research was conducted in the absence of any commercial or financial relationships that could be construed as a potential conflict of interest.

## Publisher's note

All claims expressed in this article are solely those of the authors and do not necessarily represent those of their affiliated organizations, or those of the publisher, the editors and the reviewers. Any product that may be evaluated in this article, or claim that may be made by its manufacturer, is not guaranteed or endorsed by the publisher.
